# The Novel HLA Class II Alleles DRB1*15:03:06, DQB1*03:01:69 and DPB1*04:01:95 Identified in Brazilian Donors

**DOI:** 10.1111/tan.70680

**Published:** 2026-03-24

**Authors:** Augusto Cesar Soares dos Santos Júnior, Cintia Keilla Fabreti de Oliveira, Marcelo Iraja Mion, Lucas Rafael da Silva, Raquel Aparecida Fabreti‐Oliveira

**Affiliations:** ^1^ Postgraduate Program in Health Sciences, Faculty of Medical Sciences of Minas Gerais Belo Horizonte Minas Gerais Brazil; ^2^ Department of Nephrology Hospital das Clínicas EBSERH, Federal University of Minas Gerais Belo Horizonte Minas Gerais Brazil; ^3^ Department of Molecular Biology IMUNOLAB, Laboratory of Histocompatibility Belo Horizonte Minas Gerais Brazil; ^4^ Department of Molecular Biology Xmol Laboratory, Biometrix Diagnóstica Curitiba Paraná Brazil

**Keywords:** Brazilian population, HLA class II, new allele, NGS, REDOME

## Abstract

Identification of the novel HLA alleles DRB1*15:03:06, DQB1*03:01:69 and DPB1*04:01:95 in Brazilian individuals.

HLA class II polymorphism plays a central role in immune recognition and donor–recipient compatibility in solid organ and haematopoietic stem cell transplantation. Continuous identification of novel HLA alleles is essential to maintain the accuracy of reference databases and to support high‐resolution matching strategies, particularly in genetically admixed populations such as Brazil [1].

In recent years, the HLA‐DQB1 locus has assumed an increasingly influential role in organ allocation in Brazil, transitioning from an ancillary marker to a key determinant in deceased‐donor allocation [2]. Accumulating evidence indicates that anti‐HLA‐DQB1 antibodies are strongly associated with antibody‐mediated rejection and reduced allograft survival, either independently or in conjunction with HLA‐DRB1 mismatches [3]. In this context, national allocation policies have standardised a recipient scoring system based on HLA‐DRB1 and HLA‐DQB1 identity, representing a paradigm shift aligned with international evidence and the immunogenetic profile of the Brazilian population, enabling more accurate allocation decisions, improved virtual crossmatch reliability and enhanced long‐term graft outcomes [2].

HLA alleles arise through diverse genetic mechanisms, reflecting the high degree of polymorphism characteristic of HLA loci [4]. According to the IPD‐IMGT/HLA Database version 3.63.1 (February 2026), a total of 43,758 HLA alleles have been described, of which 13,904 are HLA class II alleles. Among these, 3924 correspond to the HLA‐DRB1 locus, 3022 to HLA‐DQB1 and 3075 to HLA‐DPB1 [5]. In this report, we describe three novel HLA class II alleles, HLA‐DRB1*15:03:06, HLA‐DQB1*03:01:69 and HLA‐DPB1*04:01:95, identified in volunteer donors from the Brazilian Registry of Volunteer Bone Marrow Donors (REDOME). All donors provided written informed consent for genetic testing and data use in accordance with national regulations.

High‐resolution HLA typing was initially performed using next‐generation sequencing (NGS) with the AllType NGS 11 loci kit (One Lambda, Canoga Park, CA, USA) on the Ion Torrent S5 platform (Thermo Fisher Scientific, Waltham, MA, USA). Sequence data were analysed using TypeStream Visual software version 3.1.0 (One Lambda). To confirm the presence of the novel alleles HLA‐DQB1*03:01:69 and HLA‐DPB1*04:01:95, an independent typing was performed using the Sequence‐Based Typing (SBT) method with the SeCore HLA kit (One Lambda) on an ABI 3730 Genetic Analyzer (Applied Biosystems, Foster City, CA, USA). Resulting sequences were analysed using uType software version 7.3 (One Lambda). In addition, all three alleles were confirmed by NGS on a second platform, the DNBSEQ‐G99 (MGI, Shenzhen, China), using the same AllType NGS kit, ensuring concordant results across technologies.

Each novel allele differs from its closest known allele by a single synonymous nucleotide substitution, resulting in no amino acid change in the encoded HLA molecule. Specifically, HLA‐DRB1*15:03:06 differs from HLA‐DRB1*15:03:01 by a synonymous substitution at nucleotide position 732 (codon 215; GCC → GCT). HLA‐DQB1*03:01:69 differs from HLA‐DQB1*03:01:01 by a substitution at nucleotide position 519 (codon 141; GGC → GGA), and HLA‐DPB1*04:01:95 differs from HLA‐DPB1*04:01:01 by a substitution at nucleotide position 288 (codon 67; GAG→GAA). Extended HLA typing and GenBank accession numbers are provided in Table 1.

**TABLE 1:** Characteristics of novel HLA‐DRB1, ‐DQB1 and ‐DPB1 alleles identified in three Brazilian individuals.

Novel HLA allele	Ethnic group	HLA typing	GenBank accession number	Most similar HLA allele	Nucleotide position changed	Codon changed	Codon changes
DRB1*15:03:06	African Decent	A*33:01:01, 68:01:01 B*14:02:01, 53:01:01 C*04:01:01, 08:02:01 DRB1*07:01:01, 15:03:06 DRB4*01:03:01 DRB5*01:01:01 DQA1*01:02:01, 02:01:01 DQB1*02:02:01, 06:02:01 DPA1*02:01, 02:01 DPB1*01:01:01, 01:01:01	PQ882775	DRB1*15:03:01	732	215.3	GCC to GCT
DQB1*03:01:69	Hispanic	A*02:01:01, 03:01:01 B*44:03:01, 51:01:01 C*02:02:02, 16:01:01 DRB1*04:01:01, 15:01:01 DRB4*01:03:01 DRB5*01:01:01 DQA1*01:02:01, 03:03:01 DQB1*03:01:69, 06:03:01 DPA1*01:03:01, 01:03:01 DPB1*04:HJMR, 04:FNVS	PQ882776	DQB1*03:01:01	519	141.3	GGC to GGA
DPB1*04:01:95	African Decent	A*03:01:01, 03:01:01 B*18:01:01, 50:01:01 C*04:01:01, 05:01:01 DRB1*03:01:01, 11:02:01 DRB3*02:02:01, 02:10 DQA1*05:01, 05:05 DQB1*02:01:01, 03:19:01 DPA1*01:03:01, 01:03:01 DPB1*02:01:02, 04:01:95	PQ882777	DPB1*04:01:01	288	67.3	GAG to GAA

The name HLA‐DRB1*15:03:06 was officially assigned by the WHO Nomenclature Committee for Factors of the HLA System in February 2025, while the names HLA‐DQB1*03:01:69 and HLA‐DPB1*04:01:95 were assigned in March 2025, following the established nomenclature policies. This follows the agreed policy that, subject to the conditions stated in the most recent Nomenclature Report, names will be assigned to new sequences as they are identified. Lists of such new names will be published in the following WHO Nomenclature Report [6].

## Author Contributions

**Augusto Cesar Soares dos Santos Júnior:** data analysis, manuscript writing and manuscript review. **Cintia Keilla Fabreti de Oliveira:** technical work, data analysis and manuscript review. **Marcelo Iraja Mion:** data interpretation and contribution to manuscript drafting. **Lucas Rafael da Silva:** data interpretation and contribution to manuscript drafting. **Raquel Aparecida Fabreti‐Oliveira:** sequence submission and manuscript review. All authors have read and approved the final manuscript.

## Funding

The authors have nothing to report.

## Conflicts of Interest

The authors declare no conflicts of interest.

## Data Availability

The data that support the findings of this study are openly available in the IPD‐IMGT/HLA database at https://www.ebi.ac.uk/ipd/imgt/hla/alleles/.
